# Whole-genome sequencing of non-typeable *Haemophilus influenzae* isolated from a tertiary care hospital in Surabaya, Indonesia

**DOI:** 10.1186/s12879-024-09826-8

**Published:** 2024-10-03

**Authors:** Made Ananda Krisna, Lindawati Alimsardjono, Korrie Salsabila, Naritha Vermasari, Wa Ode Dwi Daningrat, Kuntaman Kuntaman, Odile Barbara Harrison, Martin Christopher James Maiden, Dodi Safari

**Affiliations:** 1 Eijkman Research Centre for Molecular Biology, National Research and Innovation Agency, Cibinong, West Java Indonesia; 2 Department of Biology, University of Oxford, Oxford, UK; 3 Department of Clinical Microbiology, Dr. Soetomo Academic General Hospital, Surabaya, Indonesia; 4 Centre for Genomic Pathogen Surveillance, Nuffield Department of Clinical Medicine, University of Oxford, Oxford, UK; 5 Nuffield Department of Population Health, University of Oxford, Oxford, UK; 6 Graduate School of Medical and Pharmaceutical Sciences, Chiba University, Chiba, Japan

**Keywords:** *Haemophilus influenzae*, Invasive disease, Whole-genome sequencing, Population genetics, Indonesia

## Abstract

**Background:**

*Haemophilus influenzae* causes life-threatening invasive diseases such as septicaemia and meningitis. Reports on circulating *H. influenzae* causing invasive disease in lower-middle income settings, including Indonesia, are lacking. This study describes the serotype distributions and whole-genome sequence (WGS) data of *H. influenzae* isolated from hospitalized patients at Soetomo Hospital, Surabaya, Indonesia.

**Methods:**

*H. influenzae* isolates were isolated from blood and pleural fluid specimens and identified using culture-based and molecular methods, followed by serotyping and WGS using RT‒PCR and Illumina MiSeq, respectively. Sequencing reads were assembled, and further analyses were undertaken to determine the genomic content and reconstruct the phylogeny. A second dataset consisting of publicly available *H. influenzae* genomes was curated to conduct phylogenetic analyses of isolates in this study in the context of globally circulating isolates.

**Results:**

Ten *H. influenzae* isolates from hospitalized patients were collected, and septicaemia was the most common diagnosis (*n*=8). RT‒PCR and WGS were performed to determine whether all the isolates were nontypeable *H. influenzae* (NTHi). There were four newly identified STs distributed across the two main clusters. A total of 91 out of 126 virulence factor (VF)-related genes in *Haemophilus* sp. were detected in at least one isolate. Further evaluation incorporating a global collection of *H. influenzae* genomes confirmed the diverse population structure of NTHi in this study.

**Conclusion:**

This study showed that all *H. influenzae* recovered from invasive disease patients were nonvaccine-preventable NTHi isolates. The inclusion of WGS revealed four novel STs and the possession of key VF-associated genes.

**Supplementary Information:**

The online version contains supplementary material available at 10.1186/s12879-024-09826-8.

## Introduction

*Haemophilus influenzae*, a pleomorphic gram-negative coccobacillus, frequently resides within the upper respiratory tract and is a common etiological agent of invasive and noninvasive bacterial infections [20]. *Haemophilus influenzae* type b (Hib) was the most prevalent cause of bacterial meningitis in children aged <5 years prior to the introduction of the Hib-conjugate vaccine [36]. In the postvaccination era, the prevalence of Hib disease has greatly decreased [36], however, nontypeable *H. influenzae* (NTHi) has emerged as the predominant cause of *H. influenzae* invasive disease [17, 22]. This pattern is observed across different regions worldwide. Reports from the European Centre for Disease Prevention and Control (ECDC) showed that most *H. influenzae* invasive disease cases were attributed to NTHi (78%) or non-Hib encapsulated isolates (13%) [21, 45]. According to a surveillance program in South Africa in 2018, 64% of invasive *H. influenzae infections* were due to NTHi [27]. Additionally, data from the United States indicated that NTHi has the highest incidence and case fatality rates (CFRs) in the US compared to Hib and non-Hib encapsulated serotypes [39]. Globally, invasive NTHi infection had a greater CFR (17–21%) than invasive Hib infection [37].

The Hib vaccine was introduced in the routine childhood immunization schedule in Indonesia in 2013 [31]. At the time of writing (November 2023), there was a relative lack of data on invasive *H. influenzae* infection post-Hib vaccination from the WHO South East Asia Region (SEARO), including Indonesia [37]. This scarcity of data was partly due to the limited capacity of microbiology laboratories to identify and isolate fastidious organisms. As part of a US Centers for Disease Control and Prevention (CDC)-funded capacity-building project aiming to improve the detection of fastidious organisms, including *H. influenzae*, this study is the first to describe the serotype distribution and population genomics of *H. influenzae* isolated from hospitalized patients in Surabaya, Indonesia, in 2019.

## Methods

### *Haemophilus* influenzae isolation and identification

This study used archived isolates and data from a previous project, in which routine sterile-site specimens were collected at Dr. Soetomo Academic General Hospital, a tertiary referral hospital in Surabaya, Indonesia, from January to December 2019. The specimens were inoculated into blood culture bottles and incubated in a BACTEC Incubator (BD BACTEC FX) per the manufacturer’s protocol. Positive culture bottles were streaked onto supplemented chocolate agar and incubated at 37°C with 5% CO_2_. Suspected *H. influenzae* colonies were characterized as having a non-haemolytic, opaque to gray color, creamy texture with a pungent indole smell. All suspected colonies were stored in STGG media and frozen at -80°C. The isolates were shipped to the Eijkman Institute for Molecular Biology (Jakarta, Indonesia) on dry ice for further identification by Gram staining and X&V-dependent tests [32]. Real-time PCR (RT‒PCR) for *hpd* gene detection was employed to distinguish *H. influenzae* from *H. hemolyticus* and for serotyping (Additional File 1: Supplementary Table 1) [43]. The confirmed isolates were subjected to serotyping by RT‒PCR [28, 46].

### Whole-genome sequencing and analyses

#### Whole-genome sequencing (WGS)

Bacterial DNA was extracted using the DNeasy Blood and Tissue® Kit according to the manufacturer’s protocol (Qiagen, Carlsbad, CA, USA). The Nextera XT DNA Library Prep® kit was used for library preparation before paired-end WGS (2x250) on a MiSeq (Illumina, San Diego, CA, USA) according to the manufacturer’s instructions. FastQ reads were evaluated for quality and trimmed using FastQC v.0.11.9 and Trimmomatic v.0.32 pipeline [2, 6]. Draft genomes were assembled utilizing the SPAdes v.3.15.5 toolkit with the setting optimized for 2x250 bp reads [30]. Quality assurance and quality control (QA/QC) for draft genome assemblies were verified using QUAST. Statistics for assembly quality (number of contigs, genome length, GC content, N50, and L50) are reported in Additional File 1: Supplementary Table 2 [16].

#### Genome annotation and characterization

Draft genome annotation was performed with Prokka v1.14.6 using default settings. To evaluate the assembly and annotation quality further, one representative of the clinical isolates (Genome ID 1) was aligned to the NCBI *H. influenzae* reference genome (NZ_CP007470.1) using Mauve. The draft genome contigs were reordered based on the alignment and visualized using Genovi and Proksee to mark the positions of important loci (i.e., MLST and rMLST loci) and GC skews in comparison to the reference genome.

Two additional specific annotation steps were employed for the capsule region and genes encoding virulence factors (VFs). The former was performed with the Hicap suite, an *in silico* algorithm for predicting capsule type [44]. The annotation of VF-related genes was performed based on BLAST results from the Virulence Factor Database (VFDB) [24, 29]. Virulence factors are defined as “gene products that enable a microorganism to: 1) colonize a host niche,2) proliferate,and, 3) cause tissue damage or systemic inflammation” [24], [8]. In the VFDB, VF-related genes for *Haemophilus* sp. were curated from several reference genomes: *H. influenzae* 86-028NP (NC_007146), PittEE (NC_009566), PittGG (NC_009567), Rd KW20 (NC_000907), *H. ducreyi* 35000HP (NC_002940), *H. somnus* 129T (NC_008309) and 2336 (NC_010519). The first three *H. influenzae* reference genomes were NTHi.

#### Sequence typing profile assignment

Genome assemblies and relevant, deidentified isolate characterization data were uploaded to the PubMLST website (http://pubmlst.org) [19]. All the genomes were evaluated for definitive species identification and potential contamination by characterizing the ribosomal multilocus sequence typing (rMLST) profile. rMLST is a typing method that unifies bacterial genealogy and typing by cataloging variations in 53 bacterial ribosome protein subunit (*rps*) genes. This approach is capable of categorizing bacterial sequences into taxonomic groups at all hierarchical levels [18]. The seven-locus MLST profile and clonal complex (CC) were automatically assigned on the PubMLST website.

#### Phylogenetic analysis

The genomic diversity of the isolates in the study was evaluated with phylogenetic analyses. PIRATE was used to generate a core-genome alignment from annotated draft genomes [5, 34]. A maximum likelihood (ML) tree was constructed utilizing RaXML v8 and ClonalFrameML to account for recombination events [13, 40]. The resulting tree was visualized and annotated with Microreact [4]. Analyses were implemented on two datasets. The first dataset consisted of ten *H. influenzae* genomes isolated from clinical samples. The second dataset included 506 high-quality, publicly available NTHi genomes from the PubMLST database (https://pubmlst.org/organisms/haemophilus-influenzae) in addition to the ten genomes from this study to evaluate the diversity in the context of previously published genomes (Additional File 1: Supplementary Figure 1 and Additional File 2).

## Results

### All 10 *H. influenzae* isolates causing invasive diseases at Dr. Soetomo Hospital were nontypeable

Ten *H. influenzae* isolates from ten patients were identified from sterile site specimens (Table 1). All the isolates were positive for the *hpd* gene and negative for capsule-encoding genes. These RT‒PCR-based identification and serotyping results were fully concordant with the WGS-based analyses. *H. influenzae* species identification was confirmed based on the rMLST score without any evidence of contamination (Additional File 1: Supplementary Table 3). This score represented the percentage of *rps* gene variation detected in a particular genome supported by the preexisting variation from genomes with a known species [18]. Prediction with the Hicap suite also confirmed that all the isolates were NTHi [44], [11].

**Table 1:** Demographic and basic clinical characteristics of patients with invasive *H. influenzae* infection at Soetomo Hospital from January – December 2019

**No** **(Genome ID)**	**Age group**	**Sex**	**Specimen type**	**Month of specimen collection**	**Place of specimen collection**
1	geriatric	female	blood	March	In-patient wards at Soetomo Hospital
2	geriatric	male	blood	April	
3	geriatric	female	blood	April	
4	paediatric	female	blood	May	
5	adult	male	pleural fluid	June	
6	adult	female	blood	July	
7	geriatric	male	pleural fluid (empyema)	August	
8	adult	male	bacteraemia	January	
9	paediatric	female	blood	December	
10	paediatric	female	blood	January	

**Fig. 1:**
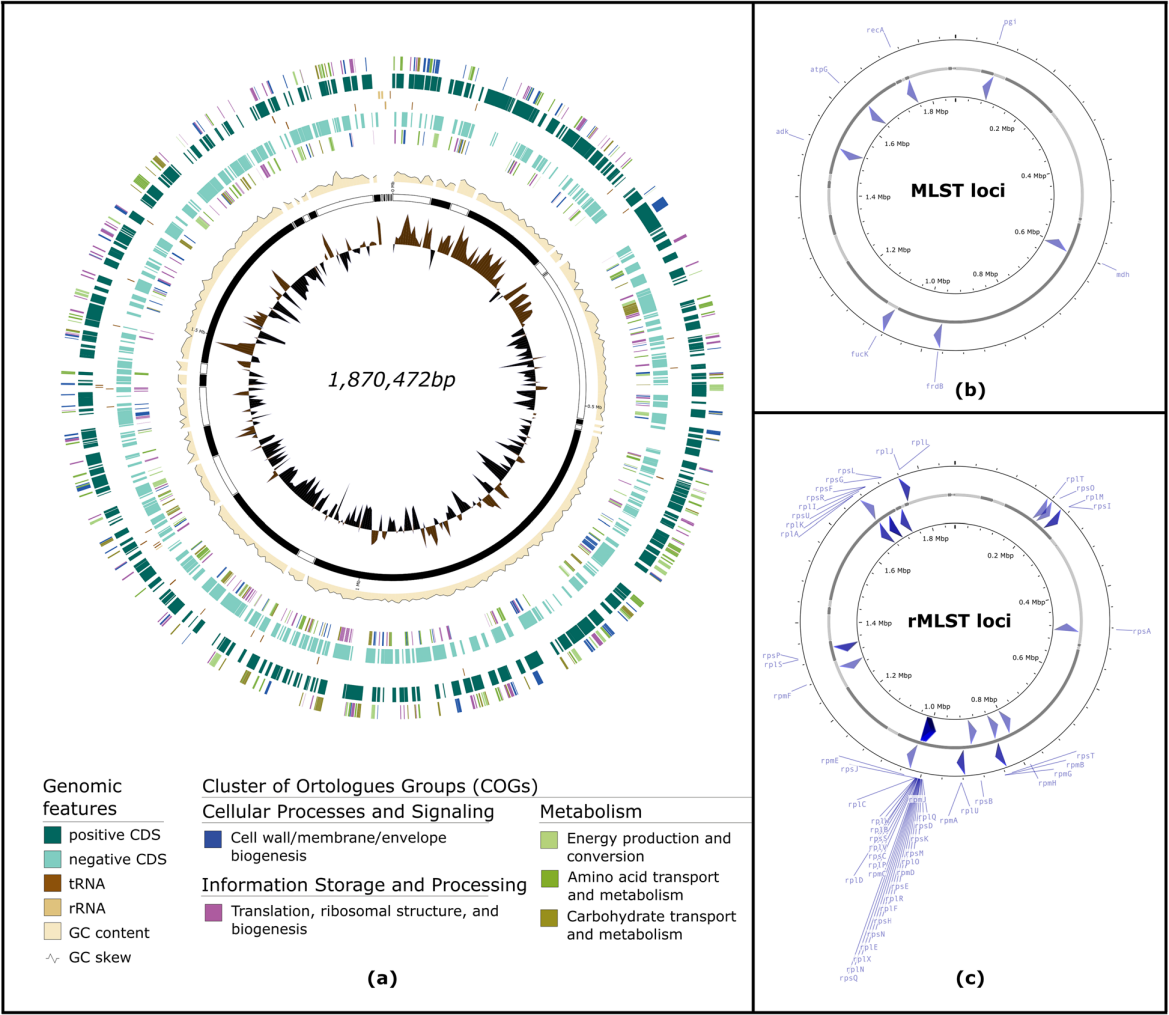
Circular schematic representation of draft *H. influenzae* genome isolated from invasive clinical isolates (Genome ID 1). The draft assembly was aligned to the complete reference genome H. influenzae 477 (NZ_CP007470.1) and its contigs were re-ordered based on the alignment using Mauve. The annotated draft genome was visualized as a circular genome using Genovi and Proksee, genome visualizers for bacteria and archaea. All CDSs were annotated and those belonging to the five most frequent clusters of orthologous groups (COG) were indicated (**a**). The position of the 7 (**b**) and 53 loci (**c**) in the MLST and rMLST scheme, respectively, were also shown

All ten *H. influenzae* draft genomes passed the QA/QC step (Additional File 1: Supplementary Table 2). As a further evaluation of the assembly and annotation quality, one of the ten *H. influenzae* genomes (Genome ID 1) was visualized schematically as a circular genome (Fig. 1). The pattern of GC skewness and position of the 7 MLST and 53 rMLST loci visualized from the draft genome highly resembled those of the reference genome (Additional File 2: Supplementary Figure 2 and 3).

Each of the ten isolates possessed different STs with four newly identified STs (STs 2571, 2579, 2576, and 2563). STs 2576 and 2563 were not part of any CCs, while STs 2571 and 2579 belonged to the CC 746 and CC 3, respectively. Among the six isolates with previously known STs, four were part of the CC 107 (STs 485, 503, 819, and 2036) with the remaining two belonged to CC 396 (ST 695) and CC 11 (ST 103).

### Two distinct clusters were observed among the 10 *H. influenzae* genomes

**Fig. 2:**
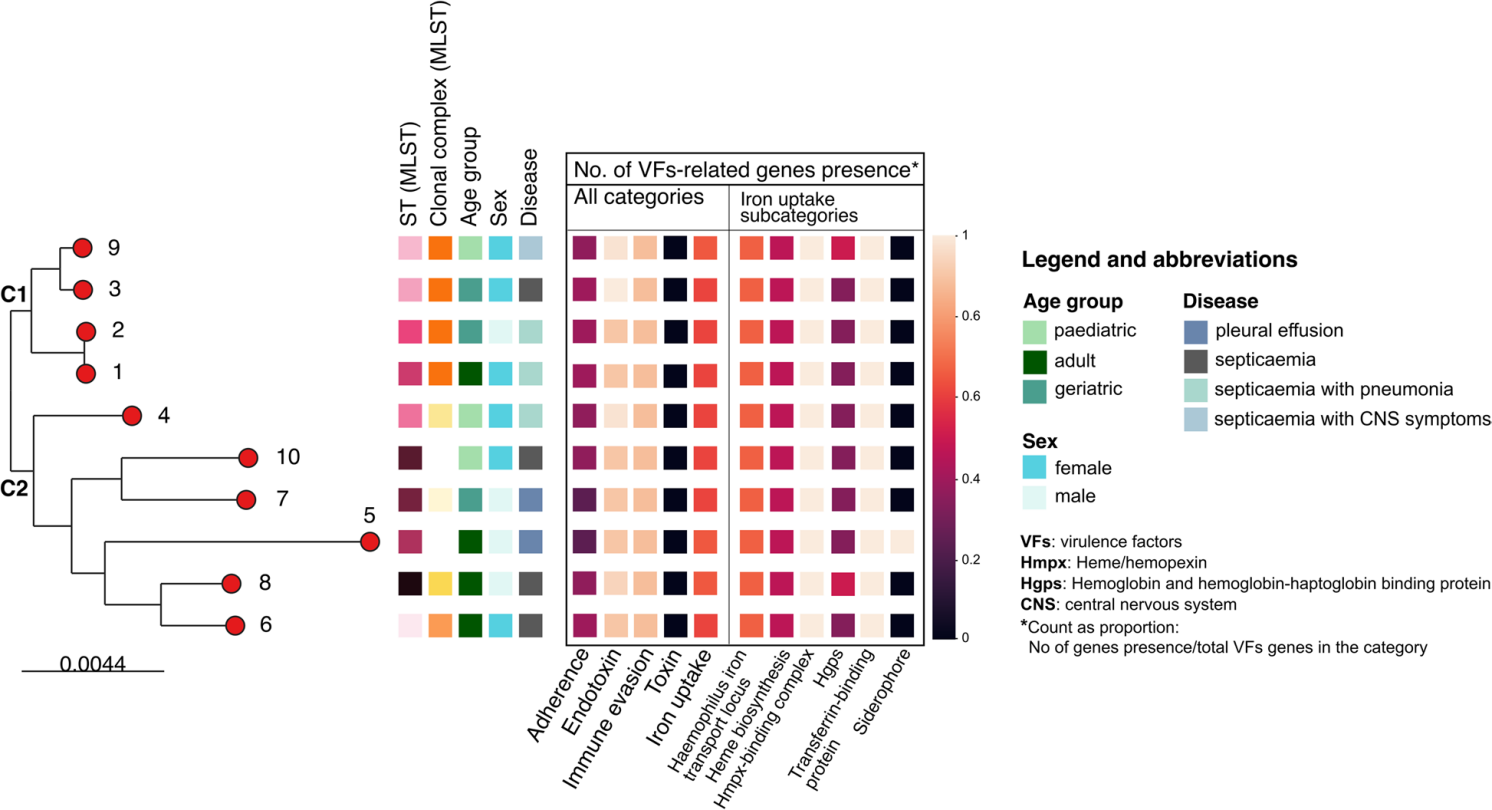
Maximum-likelihood phylogenetic tree of core-genome alignment of ten *H. influenzae* isolated from hospitalized patients in Surabaya, Indonesia from January to December 2019. Numbers alongside the nodes were genome IDs, as shown in Table 1. The tree was annotated with metadata blocks (from left to right): sequence-type, clonal group, age group, sex, and disease. The metadata blocks in the black square were the proportion of VFs-related genes present in each genome for each VF category (i.e. 1 meaning all VF-s genes in a category were present in a particular genome). The same depiction was generated for the “iron uptake” subcategories

According to the pangenome analysis of ten annotated *H. influenzae* genomes, there were 2,384 genes in total, 1,424 of which were shared. Subsequently, the core genome alignment from the analysis was utilized to construct an ML phylogenetic tree, which inferred their genetic relatedness (Fig. 2).

Two main clusters, C1 and C2, were observed following phylogenetic analyses. All four isolates in C1 belonged to the same CC, the ST-107 complex, with short branch lengths. For C2, no members in this cluster belonged to the same CC, and each had a long branch from the most recent common ancestor. Additionally, all four isolates with newly identified STs were part of C2.

Out of the four isolates clustered in C1, two originated from geriatric (≥ 60 years old) patients, one from an adult (18-60 years old), and one from a paediatric patient (< 18 years old). Only one of these patients was male. There was a total of four isolates from adult patients, three of which were grouped in C2 [35]. In contrast to C1, C2 had an equal number of isolates from male and female patients (*n* = 3 each). Although the total number of isolates was small, all patients with pleural effusion (*n* = 2) belonged to C2. Additionally, two out of three patients with both septicaemia and pneumonia were closely clustered within one of the C1 branches (Fig. 2).

### The majority of well-defined virulence factor-related genes were present in the ten *H. influenzae* genomes

**Fig. 3:**
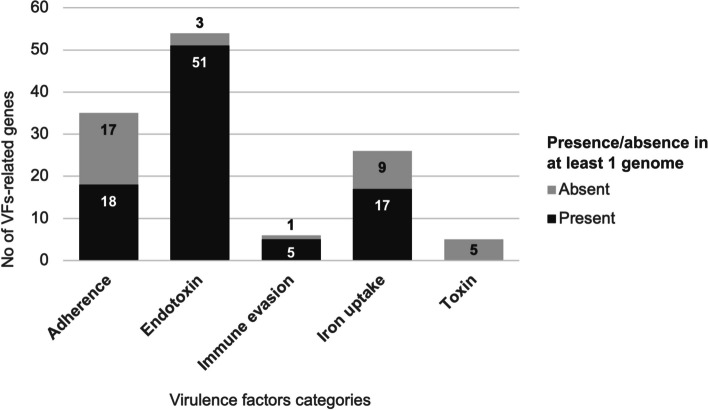
The number of virulence-associated genes present in at least one *H. influenzae* isolated from hospitalized patients in Surabaya, Indonesia from January to December 2019, grouped by virulence factors (VFs) categories

Among the 126 genes associated with known or predicted VFs in the *Haemophilus* sp. reference genome, 91 (72%) were present in at least one of the Indonesian genomes (Additional File 3). Among the 91 VF-related genes, more than half (*N*=51) were involved in lipooligosaccharide (LOS) production; only three LOS-associated genes, which encode glycosyltransferase family 52 protein (*orfO*), phosphomannomutase (*yhxB/manB*), and hypothetical protein (*lex2A*), present in all the NTHi reference genomes were absent in our collection of genomes (Fig. 3). A total of 73/91 (80.2%) VF-related genes were shared by all the genomes in the present study.

The presence of VF-related genes was also assessed in the context of the phylogenetic analyses (Fig. 2). There were a consistent number of genes present for each VF category in both clusters; however, there were two C2 isolates (Genome IDs 5 and 7) with a lower number of total adherence-associated genes. These isolates did not possess any *hmwA* or *hmwB* genes (Additional File 1: Supplementary Figure 4). The two isolates were detected in two patients with pleural effusion but without septicaemia. On the other hand, the third isolate obtained from a patient with septicaemia (Genome ID 10) without the *hmwA/B* gene had four additional adherence-related genes, *hifA/B/C/D,* which were not present in the rest of the isolates in the dataset (Additional File 1: Supplementary Figure 4).

In addition to the 126 VF-related genes commonly identified in *Haemophilus* sp., a virulence gene originating from *Acinetobacter baumannii*, *basG*, was detected in one genome (Genome ID 5)*.* This gene encodes histidine decarboxylase, an enzyme responsible for acinetobactin biosynthesis, which functions as a siderophore [26]. There was no prior evidence of the gene being present in several *Haemophilus* sp. reference genomes. Therefore, the isolate was likely to have acquired this gene via horizontal gene transfer (HGT).

### Globally circulating *H. influenzae* show a diverse population structure, with each *H. influenzae* genome from Soetomo Hospital located across the phylogeny

A total of 506 publicly available NTHi genomes were chosen to evaluate the 10 genomes from this study in the context of the global NTHi population structure. The workflow for choosing these public genomes is depicted in Additional File 1: Supplementary Figure 1. Most (375/506, 74%) genomes originated from Europe, 108 (21%) from North America, and the rest from Oceania (11, 2.2%), Africa (8, 1.5%), and Asia (4, 0.8%). A total of 405 genomes (80%) had associated provenance data of the specimen type from which they were isolated, with the most prevalent being blood (28%), sputum (27%), nasal/nasopharynx swabs (13%), and cerebrospinal fluid (11%). (Additional File 2)

**Fig. 4:**
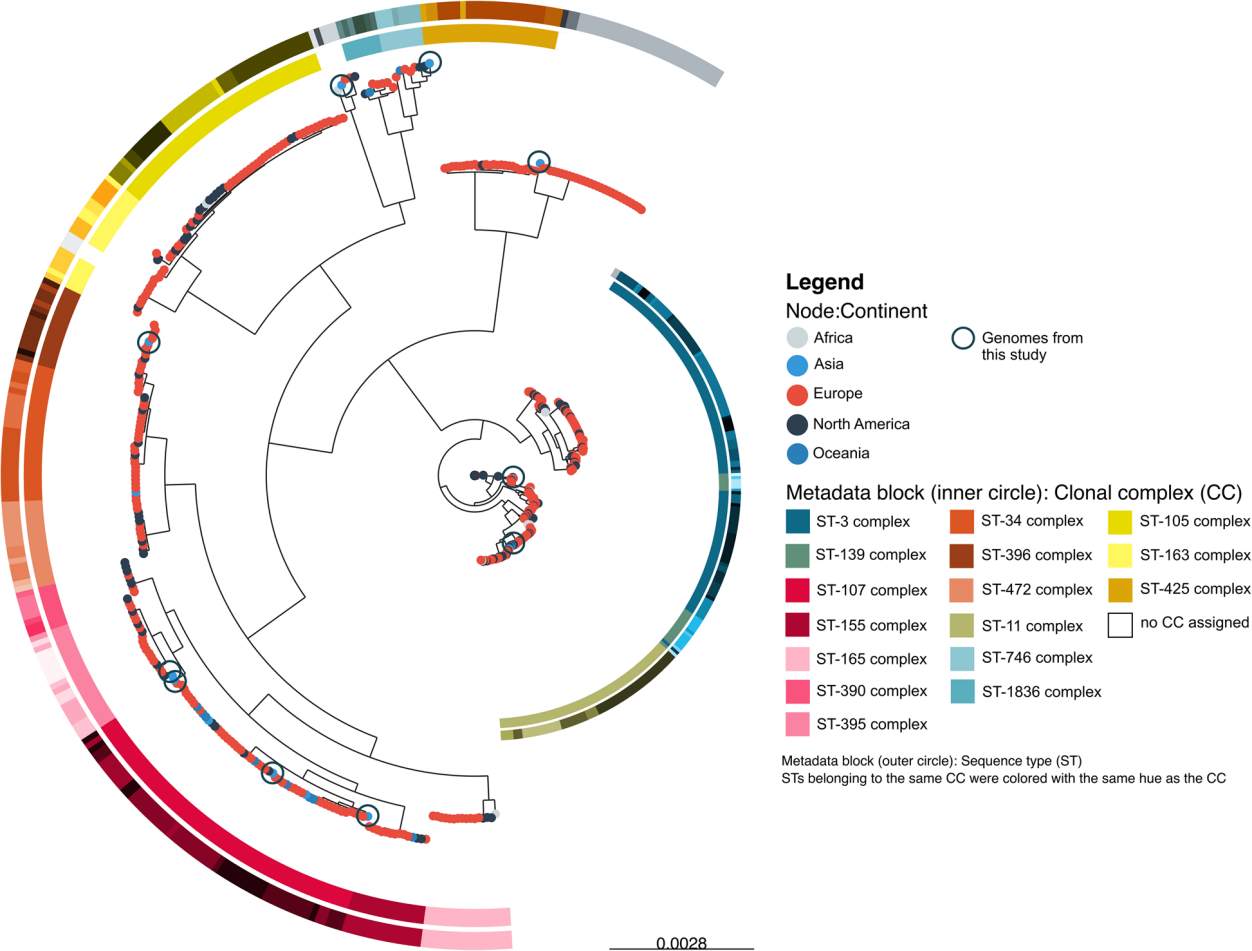
The population structure of 10 invasive NTHi isolates from Surabaya, Indonesia reflects the population structure of the selected global NTHi genomes collection. The ML tree was constructed based on the core genome alignment and had been corrected for recombination events. The nodes were coloured based on continents, while the inner and outer metadata blocks were CC and ST, respectively. Encircled nodes were the genomes from this study

Phylogenetic analysis generated from the core genome nucleotide alignment was performed for a total of 516 genomes. There was no phylogeographic structure with the Indonesian isolates sequenced in this study dispersed across the tree (Fig. 4). Two findings were noteworthy. First, five STs (STs 140, 259, 398, 423, and 914) in the ST-3 and ST-139 CCs putatively resembled ancestral genotypes, consistent with the presence of short branches on the mid-rooted tree. Additionally, a higher degree of genomic diversity was present in isolates belonging to these CCs. Second, genomes with STs not clustering with either ST-3 or ST-139 CCs showed a more clonal structure, indicative of genotypes that diverged from the ancestral state. Indonesian isolates from this study were found either close to the putative ancestral lineage or in clonal groups at the tips of the branches.

## Discussion

Although confined to one referral hospital in the country, this study is the first surveillance report of *H. influenzae* invasive disease in Indonesia. Building the capacity to isolate and conduct microbiological identification of the bacterium was essential for this project. Once capacity was in place, WGS was incorporated into the analysis to provide more insights into the population structure of circulating bacteria causing invasive disease. This approach strengthened the microbiological capacity and ensured that WGS analyses could be incorporated with high confidence due to the presence of robust sample processing.

All (10/10) *H. influenzae* isolated from sterile clinical specimens in this study were NTHi. Consistent with multiple epidemiology reports worldwide, after more than two decades of Hib vaccine implementation, NTHi is the major cause of invasive *H. influenzae* disease. The central public health laboratory in the province of Ontario, Canada, and the national referral laboratories in Paraguay and Argentina reported NTHi as the underlying type for 62.3%, 57.9%, and 44.5% of invasive *H. influenzae* disease, respectively [1, 14, 23]. However, there are unique findings in particular regions, such as *H. influenzae* type a as the dominant etiology for invasive diseases (50%) in Northwestern Ontario, Canada and the re-emergence of Hib in Argentina (41.1%) [14], [15]. These reports mostly come from higher-income settings and/or areas outside the WHO South East Asia Region (SEARO), as at the time of the writing, there were limited data on *H. influenzae* infection in the lower-middle income setting, especially in SEARO. Nevertheless, we cannot comment on the invasive *H. influenzae* disease trend in the post-Hib vaccination era in Indonesia, as this is the first-ever report of this disease in this country.

Most (7/10) patients with *H. influenzae* disease were adults, including four elderly patients (defined as individuals ≥ 60 years old, per the Indonesia Presidential Legislation for National Strategy for Older People) [3]. This finding is in accordance with the known age distribution of invasive NTHi disease in the post-Hib vaccination era. Surveillance reports from the ECDC in 2007–2014 revealed similar findings, with a median age of 58 years for patients with invasive NTHi infections [45]. In Spain, over a study period dating from 2008 to 2019 at a tertiary care center for adults, 65.8% of invasive NTHi patients were > 65 years old [7]. Data from the Queensland Public Health Microbiology Laboratory from 2001 to 2015 demonstrated similar findings, with a median age of 50.5 years [41].

Two patients with invasive NTHi disease in the present study were children under five years old, one of whom was a newborn. A South African surveillance program revealed that the incidence of invasive NTHi was highest in infants [37], and a report from Queensland revealed that 36.5% (54/148) of the cases were in this age group [10]. Additionally, 42% of under five invasive *H. influenzae* infections in Japan were detected in infants [42]. Regardless of geographical region, non-typeable *H. influenzae* has been mentioned as an emerging cause of neonatal sepsis, which was the clinical diagnosis of invasive NTHi disease in newborns in this study.

The diversity observed among the ten NTHi isolates in this study was consistent with the established knowledge of the population structure of this bacterium. Among the 5,345 isolate records in PubMLST database (accessed 13^th^ November 2023), there were fewer than 14 isolates in the database belonging to 9 of the 10 STs identified in this study. Although an underlying sampling bias cannot be excluded, the *H. influenzae* isolates recovered in this study may reflect the emergence of NTHi genotypes belonging to the ST-107 clonal complex.

The pattern that emerged in the phylogenetic analyses has been reported previously in studies evaluating the population structure of invasive NTHi isolates. Phylogenetic analysis based on core genome single nucleotide polymorphisms (SNPs), or core SNPs, of 58 invasive NTHi isolates from a single tertiary care center in Spain revealed that 30 of them shared one most recent common ancestor with short branches, while the rest displayed high genetic diversity [7]. A report from Denmark's National Surveillance Laboratory of 503 isolates also revealed a similar population structure [38]. This observation suggested that there are two distinct groups of invasive NTHi isolates based on their degree of genome diversity. However, genetic relatedness investigations of invasive clinical *H. influenzae* isolates from Queensland, Australia, did not reveal this distinction [41]. These inconsistencies may be partly caused by the use of core-SNP-based phylogenetic analyses, which rely on the use of an appropriate reference genome, rather than employing *de novo* assembled core genome alignments [33]. Nevertheless, *H. influenzae*, especially NTHi, has been described for its capacity to undergo horizontal gene transfer and recombination events, contributing significantly to diversification [25].

To date, there has been only one in-depth, complete analysis of *H. influenzae* virulence gene profiles, which included 88 invasive NTHi isolates from Portugal [29]. Similar to our findings, this study revealed that the presence of one adhesin-associated gene, *hmwA*, was almost always accompanied by *hmwB.* A similar pattern was observed for invasive NTHi isolates from Spain [7]. The *hmwA* and *hmwB* genes encode two proteins in the two-partner secretion (TPS) pathway in the type V secretion system (TVSS). The concurrent presence of *hmwA* and *hmwB* genes observed in the current and previous studies might indicate the interconnected function of their products [9]. In addition, a study reported a possible immune modulation role of these products in *H. influenzae* [12]. Although there was no further evaluation of how this characteristic relates to clinical manifestations of infection, the resulting compromised immune system could result in a more widespread infection, such as septicemia. The descriptive nature of both current and previous studies prevents any conclusion from being drawn on the relationship between virulence gene presence and clinical outcome. Further *in silico* and *in vitro* research with a more comprehensive dataset is needed to unravel the complex interplay between the virulence gene repertoire and pathogen population dynamics and its potential impact on clinical manifestations.

## Conclusion

This study characterized 10 confirmed NTHi isolates from 10 invasive disease patients in a tertiary referral hospital in Surabaya, Indonesia. The isolation and identification of fastidious bacteria were made possible through a capacity-building project and can feasibly become a part of a routine surveillance program. Integrated WGS analyses revealed four novel STs and added value to understanding the population genomics and biology of the bacterium.

## Supplementary Information

Additional file 1. Supplementary tables and figures for the article “Whole-genome sequencing of Nontypeable *Haemophilus influenzae* isolated from a tertiary care hospital in Surabaya, Indonesia”.

Additional file 2. Global dataset metadata.

Additional file 3. Virulence factors presence-absence of 10 non-typeable *Haemophilus influenzae*.

## Data Availability

The datasets supporting the conclusions of this article are included within the article and its additional files. The sequence reads were deposited in the NCBI Sequence Read Archive under the project accession number PRJNA1043690.
