# Correction: Whole-genome sequencing of non-typeable *Haemophilus influenzae* isolated from a tertiary care hospital in Surabaya, Indonesia

**DOI:** 10.1186/s12879-024-10234-1

**Published:** 2024-11-21

**Authors:** Made Ananda Krisna, Lindawati Alimsardjono, Korrie Salsabila, Naritha Vermasari, Wa Ode Dwi Daningrat, Kuntaman Kuntaman, Odile Barbara Harrison, Martin Christopher James Maiden, Dodi Safari

**Affiliations:** 1 Eijkman Research Centre for Molecular Biology, National Research and Innovation Agency, Cibinong, West Java Indonesia; 2 Department of Biology, University of Oxford, Oxford, UK; 3 Department of Clinical Microbiology, Dr. Soetomo Academic General Hospital, Surabaya, Indonesia; 4 Centre for Genomic Pathogen Surveillance, Nufeld Department of Clinical Medicine, University of Oxford, Oxford, UK; 5 Nufeld Department of Population Health, University of Oxford, Oxford, UK; 6 Graduate School of Medical and Pharmaceutical Sciences, Chiba University, Chiba, Japan

**Correction** **:** **BMC Infect Dis 24, 1097 (2024)**

**https://doi.org/10.1186/s12879-024-09826-8**

Following publication of the original article [1], it was flagged to us that an additional statement needs to be included in the Acknowledgements section (marked in bold below):

 “The archived specimen in this study was obtained from the capacity-building project funded by the Grant or Cooperative Agreement Number NU2GGH001852-03, funded by the Centers for Disease Control and Prevention. Its contents are solely the responsibility of the authors and do not necessarily represent the official views of the Centers for Disease Control and Prevention or the Department of Health and Human Services. **The genomic analyses in this study was funded by a Wellcome Trust Biomedical Resource Grant to MJCM (grant number 218205/Z/19/Z)**. The funders had no role in the study design, data collection and analysis, decision to publish, or preparation of the manuscript.”

The original article has been corrected.
