# Supplementary material for: Whole-genome sequencing of non-typeable Haemophilus influenzae isolated from a tertiary care hospital in Surabaya, Indonesia
Source: BMC Infect Dis. 2024 Oct 3;24:1097. doi: 10.1186/s12879-024-09826-8 (PMC11448046; doi:10.1186/s12879-024-09826-8)

Supplementary Tables and Figures

Supplementary Table 1. Probe and Primers for *hpd* Gene Detection and Serotyping of *Haemophilus influenzae*

| **Target gene** | **Primer or Probe Name** | **Real-time Primers and Probes Nucleotide Sequence (5' to 3')** | **Working Stock**  **Conc (µM)** | **Final Conc (nM)** | **Suggested Probe Modifications** |
| --- | --- | --- | --- | --- | --- |
| ***hpd*** | hpdF822 | GGTTAAATATGCCGATGGTGTTG | 1.25 | 100 |  |
|  | hpdR952 | TGCATCTTTACGCACGGTGTA | 3.75 | 300 |  |
|  | Pb896i | TTGTGTACACTCCGT"T"GGTAAAAGAACTTGCAC | 1.25 | 100 | 5' FAM, BHQ1 on "T", 3' SpC6 |
|  |  |  |  |  |  |
| **Serotyping** | | | | | |
| **Hia** | F261 | GGT CTG CGG TGT CCT GTG T | 1.25 | 100 |  |
| ***acsB*** | R427 | CCG GTC ATC TTT TAT GCT CCA A | 3.75 | 300 |  |
|  | Pb375i | TAA TTT TCT TGC “T”CA ATA CCG CCT TCC CA | 1.25 | 100 | 5' FAM, BHQ1 on "T", 3' SpC6 |
|  |  |  |  |  |  |
| **Hib** | F192 | TGA TGC ATT GAA AGA AGG TGT AAT TT | 3.75 | 300 |  |
| ***bcsB*** | R359 | CCT GCG GTA ATA ACA TGA TCA TAA A | 7.5 | 600 |  |
|  | Pb244i | TGT CGT GCA G”T”A GCA AAC CGT AAC CTT ACT C | 1.25 | 100 | 5' FAM, BHQ1 on "T", 3' SpC6 |
|  |  |  |  |  |  |
| **Hic** | F7667 | CAT TGG TGA TGG TTC AGT TAT TGG | 7.5 | 600 |  |
| ***ccsD*** | R7784 | TAC AGC ATT CAG CAA TAA TGG G | 3.75 | 300 |  |
|  | Pb7726i | ATT GCA “T”CG CCG CAG GAG TTC CCG | 2.5 | 200 | 5' FAM, BHQ1 on "T", 3' SpC6 |
|  |  |  |  |  |  |
| **Hid** | F2211 | CCT AAA ATA CGG ACC TAG TGC AC | 7.5 | 600 |  |
| ***dcsE*** | R2255 | CCG ATG AGA CCA AGT ATG GTT A | 1.25 | 100 |  |
|  | Pb2221i | AAC GAG C”T”A GAG CTG GTG CTG AA | 3.75 | 300 | 5' FAM, BHQ1 on "T", 3' SpC6 |
|  |  |  |  |  |  |
| **Hie** | F1523 | ACT AAA ATA TGG CCC AAA CCC AC | 7.5 | 600 |  |
| ***ecsH*** | R1589 | CCG ATG AGC CCA AGT ATG ATG A | 7.5 | 600 |  |
|  | Pb1555i | AAC GAG CAA AAG CCG G”T”G CGG AT | 2.5 | 200 | 5' FAM, BHQ1 on "T", 3' SpC6 |
|  |  |  |  |  |  |
| **Hif** | F7164 | CCC TGA AAA GCG TTG ACT TTG | 7.5 | 600 |  |
| ***bexD*** | R7313 | CCA ACT TCA GGA CCA AGT CAT TC | 3.75 | 300 |  |
|  | Pb7242i | TGC TGC TAA C”T”C AGA TGC ATC AGC TCC TT | 2.5 | 200 | 5' FAM, BHQ1 on "T", 3' SpC6 |

***Note: Thermal cycler settings***

1x: 50°C for 2min

1x: 95°C for 10min

40x:

95°C for 15sec

60°C for 1min

Supplementary Table 2. Genome assembly quality.

| **Genome ID** | **Contigs** | **Total length** | **Min** | **Max** | **Mean** | **StdDev** | **N50** | **L50** | **N90** | **L90** | **N95** | **L95** | **%GC** |
| --- | --- | --- | --- | --- | --- | --- | --- | --- | --- | --- | --- | --- | --- |
| 1 | 30 | 1870472 | 515 | 541325 | 62349 | 113699 | 244159 | 3 | 30955 | 11 | 18142 | 15 | 38.01 |
| 2 | 31 | 1818468 | 515 | 500704 | 58660 | 103574 | 171533 | 3 | 49229 | 12 | 41363 | 14 | 37.96 |
| 3 | 27 | 1779948 | 510 | 546028 | 65924 | 119951 | 250466 | 3 | 48962 | 10 | 27505 | 12 | 37.87 |
| 4 | 38 | 1774877 | 507 | 321093 | 46707 | 72812 | 137102 | 5 | 31098 | 14 | 24792 | 17 | 37.86 |
| 5 | 27 | 1814967 | 579 | 920796 | 67221 | 178031 | 920796 | 1 | 26952 | 9 | 19545 | 13 | 37.84 |
| 6 | 27 | 1899188 | 535 | 543858 | 70340 | 111590 | 155971 | 4 | 51520 | 12 | 27237 | 15 | 38.03 |
| 7 | 18 | 1830682 | 559 | 612940 | 101705 | 174556 | 433432 | 2 | 102498 | 6 | 41084 | 7 | 38.09 |
| 8 | 26 | 1853037 | 517 | 543145 | 71271 | 113361 | 134732 | 4 | 45758 | 12 | 32578 | 14 | 38.04 |
| 9 | 27 | 1776175 | 545 | 377834 | 65784 | 99765 | 240576 | 3 | 49295 | 11 | 27378 | 13 | 37.87 |
| 10 | 28 | 1856440 | 503 | 709995 | 66301 | 138874 | 234643 | 2 | 52155 | 10 | 26093 | 13 | 38.05 |

Supplementary Table 3. Species identification and contamination evaluation based on rMLST score.

| **Genome ID** | **rank** | **taxon** | **support** | **rST** | **species** |
| --- | --- | --- | --- | --- | --- |
| 1 | SPECIES | *Haemophilus influenzae* | 100% | 125160 | *Haemophilus influenzae* |
| 2 | SPECIES | *Haemophilus influenzae* | 100% | 125160 | *Haemophilus influenzae* |
| 3 | SPECIES | *Haemophilus influenzae* | 100% | 125169 | *Haemophilus influenzae* |
| 4 | SPECIES | *Haemophilus influenzae* | 100% | 154450 | *Haemophilus influenzae* |
| 5 | SPECIES | *Haemophilus influenzae* | 96% | 190900 | *Haemophilus influenzae* |
| 6 | SPECIES | *Haemophilus influenzae* | 100% | 24211 | *Haemophilus influenzae* |
| 7 | SPECIES | *Haemophilus influenzae* | 98% | 190910 | *Haemophilus influenzae* |
| 8 | SPECIES | *Haemophilus influenzae* | 100% | 190917 | *Haemophilus influenzae* |
| 9 | SPECIES | *Haemophilus influenzae* | 100% | 190918 | *Haemophilus influenzae* |
| 10 | SPECIES | *Haemophilus influenzae* | 96% | 197931 | *Haemophilus influenzae* |

Supplementary Figure 1. (a) Choosing high-quality, publicly available NTHi draft genomes from PubMLST to evaluate the diversity of the ten NTHi genomes in this study in the context of previously published genomes. Draft genomes were categorized as “high-quality” if they fulfilled the following criteria: 1) Total number of contigs < 250; 2) Total assembly length between 1,7 – 2,1 Mbps; 3) N50 > 25,000; 4) L50 < 25; and 5) No evidence of contamination as indicated from both total length and rMLST profile assignment (1). The complete dataset of these genomes is available in Supplementary File 1 (b) Minimum-spanning tree of the public NTHi genomes in and including ten genomes in the current study.


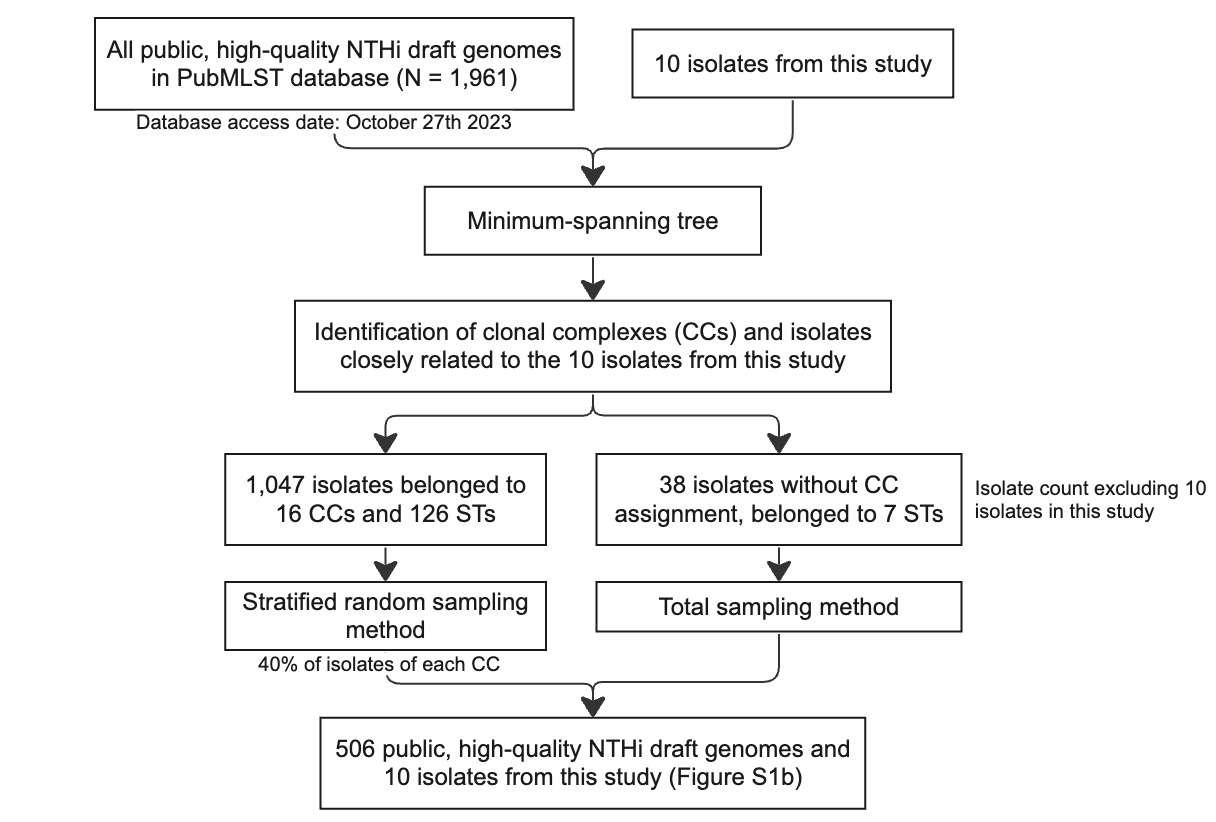

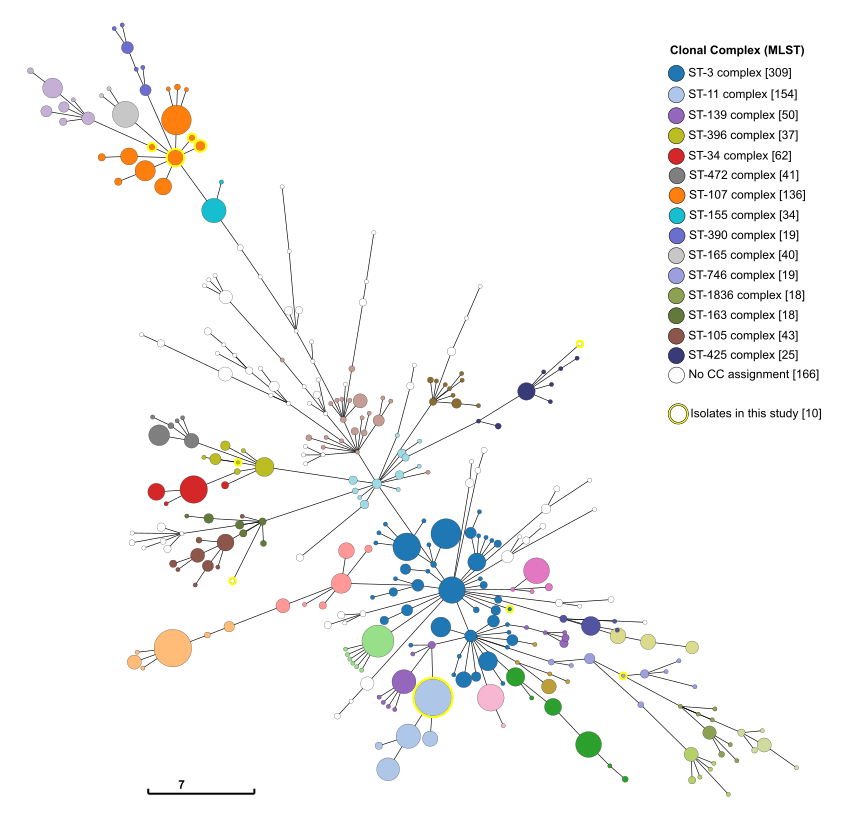


(a)

(b)

1. Tatusova T, Ciufo S, Fedorov B, O’Neill K, Tolstoy I. RefSeq microbial genomes database: new representation and annotation strategy. Nucleic Acids Res. 2014 Jan;42(Database issue):D553-559.

Supplementary Figure 2. Whole-genome alignment visualization between *H. influenzae* reference genome 477 and Genome ID 1.


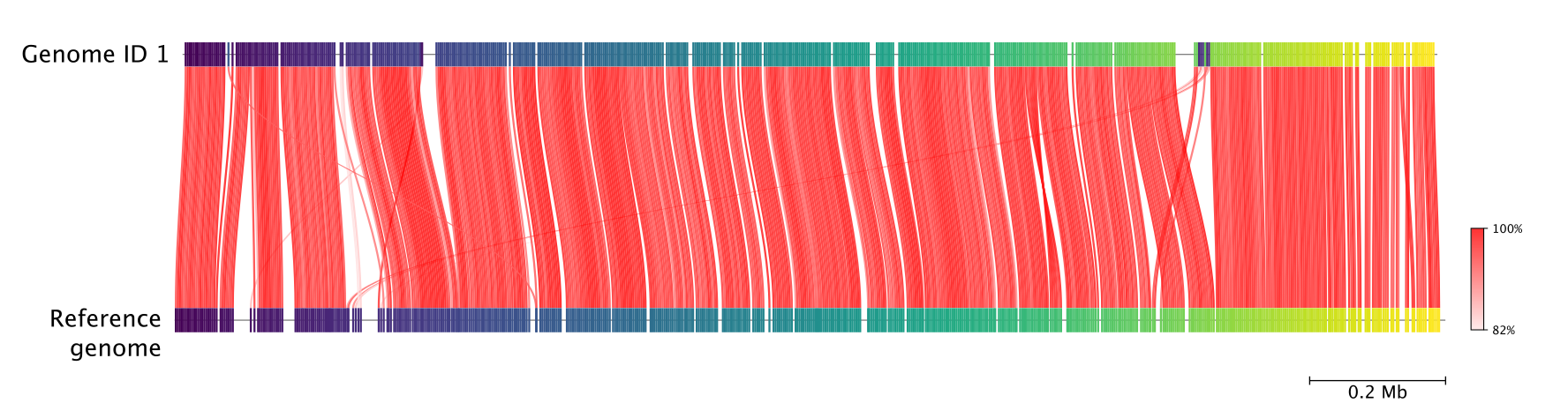


Supplementary Figure 3. *H. influenzae* reference genome 477 visualization as a circular genome, annotated with 1) CDS position and functional group; 2) GC skewness; 3) Specific position of 7 MLST loci and 53 rMLST loci.


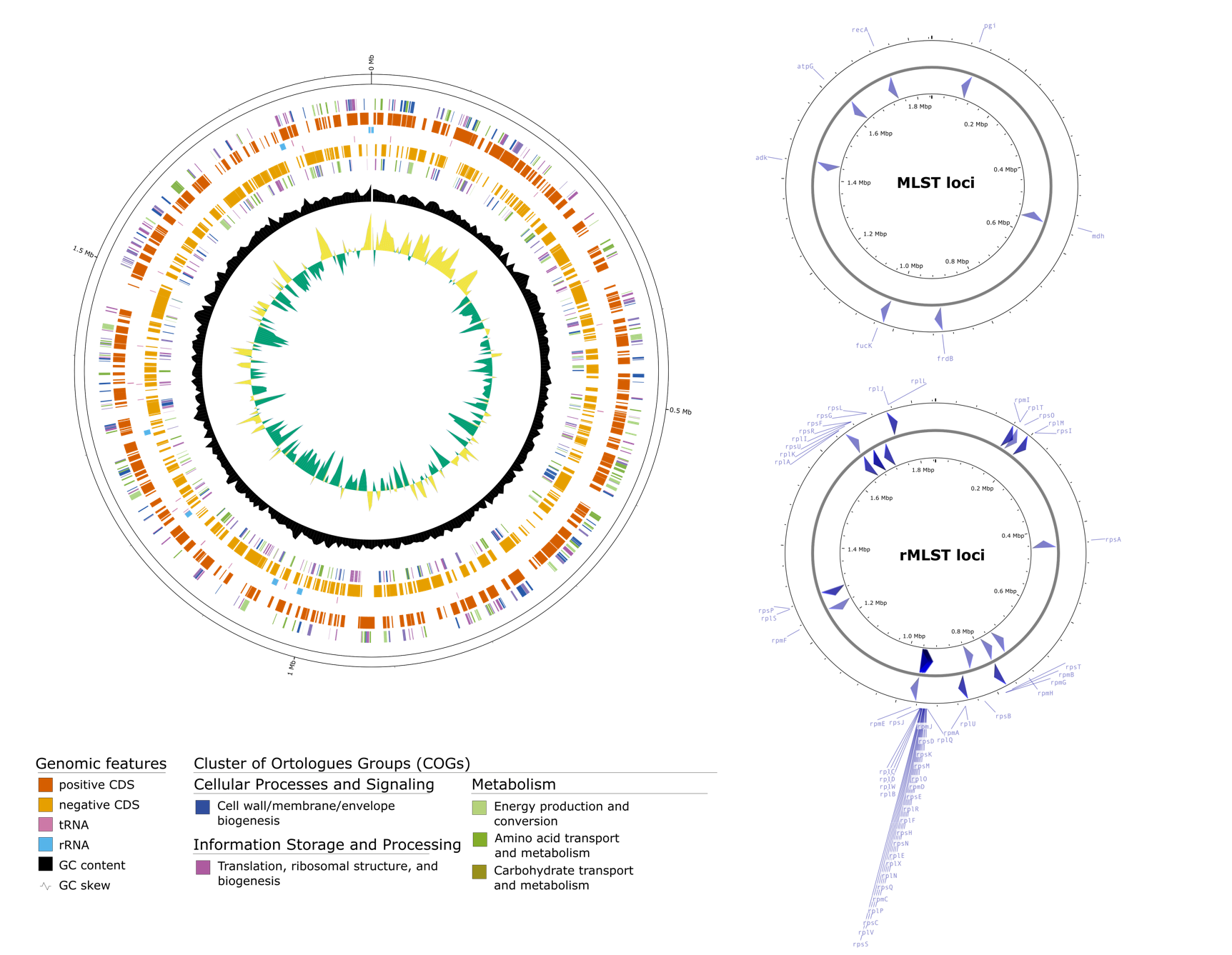


Supplementary Figure 4. Phylogeny of ten *H. influenzae* genomes from invasive disease isolates, annotated with virulence factors-related genes differentially present among the genomes.


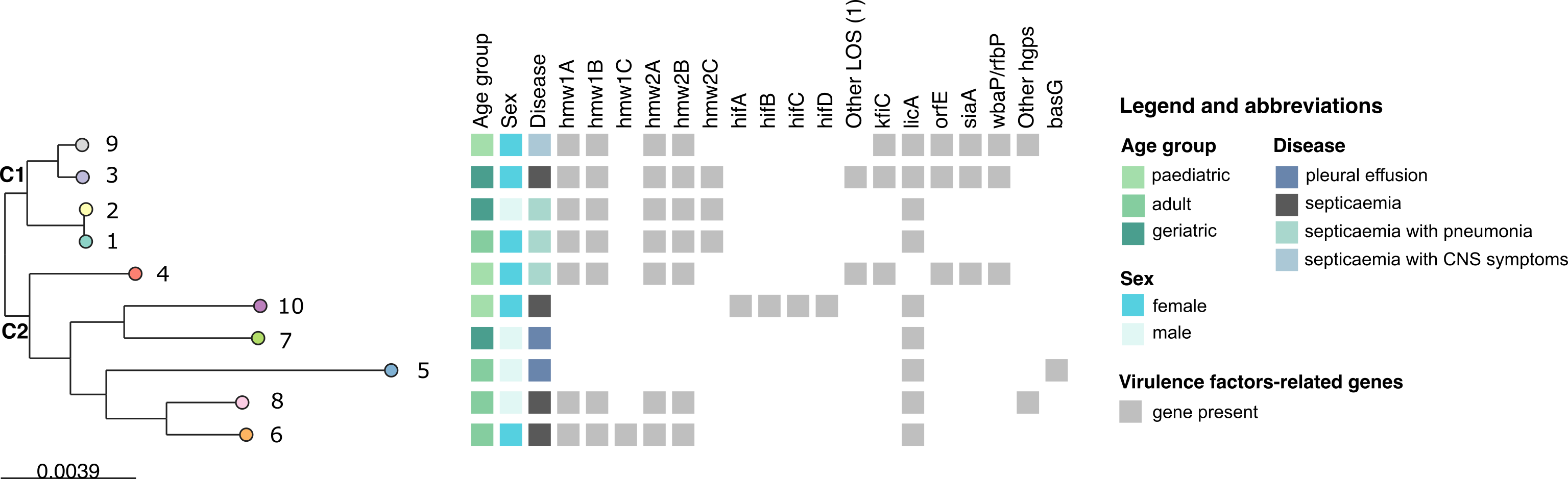

Supplement: Supplementary file 1 — Additional file 1. Supplementary tables and figures for the article “Whole-genome sequencing of Nontypeable Haemophilus influenzae isolated from a tertiary care hospital in Surabaya, Indonesia”. [file 12879_2024_9826_MOESM1_ESM.docx]
